# Bitopic Binding Mode of an M_1_ Muscarinic Acetylcholine Receptor Agonist Associated with Adverse Clinical Trial Outcomes[Fn FN3]

**DOI:** 10.1124/mol.118.111872

**Published:** 2018-06

**Authors:** Sophie J. Bradley, Colin Molloy, Christoffer Bundgaard, Adrian J. Mogg, Karen J. Thompson, Louis Dwomoh, Helen E. Sanger, Michael D. Crabtree, Simon M. Brooke, Patrick M. Sexton, Christian C. Felder, Arthur Christopoulos, Lisa M. Broad, Andrew B. Tobin, Christopher J. Langmead

**Affiliations:** The Centre for Translational Pharmacology, Institute of Molecular, Cell, and Systems Biology, College of Medical, Veterinary, and Life Sciences, University of Glasgow, Glasgow, Scotland (S.J.B., C.M., K.J.T., L.D., S.M.B., A.B.T.); Eli Lilly & Co. Neuroscience, Windlesham, Surrey, United Kingdom (C.B., A.J.M., H.E.S., M.D.C., L.M.B.); Drug Discovery Biology, Monash Institute of Pharmaceutical Sciences, Monash University, Parkville, Victoria, Australia (P.M.S., A.C., C.J.L.); and Eli Lilly & Co. Neuroscience, Indianapolis, Indiana (C.C.F.)

## Abstract

The realization of the therapeutic potential of targeting the M_1_ muscarinic acetylcholine receptor (mAChR) for the treatment of cognitive decline in Alzheimer’s disease has prompted the discovery of M_1_ mAChR ligands showing efficacy in alleviating cognitive dysfunction in both rodents and humans. Among these is GSK1034702 (7-fluoro-5-methyl-3-[1-(oxan-4-yl)piperidin-4-yl]-1*H*-benzimidazol-2-one), described previously as a potent M_1_ receptor allosteric agonist, which showed procognitive effects in rodents and improved immediate memory in a clinical nicotine withdrawal test but induced significant side effects. Here we provide evidence using ligand binding, chemical biology and functional assays to establish that rather than the allosteric mechanism claimed, GSK1034702 interacts in a bitopic manner at the M_1_ mAChR such that it can concomitantly span both the orthosteric and an allosteric binding site. The bitopic nature of GSK1034702, together with the intrinsic agonist activity and a lack of muscarinic receptor subtype selectivity reported here, all likely contribute to the adverse effects of this molecule in clinical trials. Although they impart beneficial effects on learning and memory, we conclude that these properties are undesirable in a clinical candidate due to the likelihood of adverse side effects. Rather, our data support the notion that “pure” positive allosteric modulators showing selectivity for the M_1_ mAChR with low levels of intrinsic activity would be preferable to provide clinical efficacy with low adverse responses.

## Introduction

The M_1_ muscarinic acetylcholine receptor (mAChR) has emerged as an attractive molecular target to overcome cognitive decline associated with cholinergic degeneration in Alzheimer disease (AD) (Anagnostaras et al., 2003). Activation of M_1_ mAChRs, which are abundantly expressed in the amygdala, cerebral cortex, striatum, and hippocampus (Buckley et al., 1988; Levey et al., 1995), has been reported to rescue learning and memory deficits associated with neurodegeneration in a number of mouse models (Lange et al., 2015; Puri et al., 2015; Vardigan et al., 2015; Bradley et al., 2017). Translating these promising findings to successful clinical candidates has, however, been challenging due to adverse effects associated with a lack of selectivity of orthosteric M_1_ mAChR agonists. This is exemplified by the M_1/_M_4_-preferring mAChR agonist xanomeline, which significantly improved cognitive function in patients with AD (Bodick et al., 1997) but ultimately failed due to adverse effects attributed to activation of peripheral cholinergic signaling likely through M_2_ and M_3_ mAChRs (Langmead et al., 2008).

There is, therefore, an urgent need to develop novel approaches to build selectivity into M_1_ mAChR ligands. Two related approaches to this problem have been taken: 1) the development of positive allosteric modulators (PAMs) that bind to a site that is topographically distinct from that of the endogenous ligand acetylcholine (ACh) (May et al., 2007) and 2) development of a newer generation of more selective agonists (whose mechanism of action is not always well defined) (Langmead et al., 2008). Allosteric modulators enhance ACh binding and/or signaling, the magnitude of which can vary with different degrees of positive cooperativity (Langmead and Christopoulos, 2006; May et al., 2007; Conn et al., 2009). They can also possess direct allosteric agonist activity (intrinsic activity). M_1_ mAChR allosteric modulators with high functional selectivity over other muscarinic receptor subtypes (i.e., M_2_–M_5_ mAChRs) have been reported to reverse phenotypes associated with neurodegenerative disease (Ma et al., 2009; Puri et al., 2015; Vardigan et al., 2015; Bradley et al., 2017) while showing no adverse side effects in animal models (Bradley et al., 2017).

Less well described in the literature is the development of a new generation of M_1_ mAChR agonists, which have variously been described as “ectopic” (Spalding et al., 2002), “allosteric” (Langmead and Christopoulos, 2006; Jones et al., 2008; Budzik et al., 2010), “atypical” (Lebon et al., 2009), and “bitopic” (Keov et al., 2011). With but a few exceptions (Keov et al., 2014), this broad nomenclature reflects a paucity in understanding of mechanism(s) of action; it is not clear whether some of these ligands exert true allosteric agonism (i.e., bind solely to an allosteric binding site to activate the receptor), are subtype-selective orthosteric agonists, or represent a hybrid of the two (with a pharmacophore that engages both orthosteric and allosteric binding pockets).

GSK1034702 (7-fluoro-5-methyl-3-[1-(oxan-4-yl)piperidin-4-yl]-1*H*-benzimidazol-2-one) has been described as an allosteric M_1_ mAChR agonist; it was identified from a series of benzimidazolones and reversed scopolamine-induced amnesia in rodents (Budzik et al., 2010) and had positive effects on cognitive function in humans. Unfortunately, it also induced gastrointestinal adverse effects, consistent with activation of peripheral mAChRs (Nathan et al., 2013).

Here we conduct a comprehensive pharmacological analysis of GSK1034702 and show that this molecule is not a purely allosteric ligand as previously suggested. Radioligand binding, using [^3^H]-*N*-methylscopolamine (NMS) and [^3^H]-GSK1034702, and functional inositol phosphate (IP) accumulation studies at the muscarinic M_1_ receptor reveal that GSK1034702 interacts with the orthosteric ACh binding site and likely, concomitantly, with an allosteric binding site. Importantly, this bitopic mode of action is able to mediate beneficial effects on learning and memory but the lower degree of selectivity of GSK1034702 compared with ligands that engage solely with an allosteric binding site, together with the intrinsic agonist activity reported here, may account for the adverse effects observed with this molecule in the clinic.

## Materials and Methods

### 

#### Materials.

GSK1034702 and xanomeline were synthesized by Eli Lilly (Windlesham, Surrey, UK). IP-One and extracellular signal–regulated kinase 1/2 (ERK1/2) phosphorylation assay kits were purchased from Cisbio Assays (Codolet, France). TBPB (1-[1′-(2-methylbenzyl)-1,4′-bipiperidin-4-yl]-1,3-dihydro-2*H*-benzimidazol-2-one) was obtained from Tocris Bioscience (Bristol, UK). All other chemicals and reagents were purchased from Sigma-Aldrich Company Ltd. (Dorset, UK).

#### Mouse Maintenance and Diet.

All experiments were performed under a project license from the British Home Office (United Kingdom) under the Animals (Scientific Procedures) Act of 1986. C57Bl/6J mice used in this study were purchased from Charles River (Margate, UK). Mice were fed ad libitum with a standard mouse chow and were maintained within the animal facility at least 1 week prior to experiments.

#### Fear Conditioning.

C57Bl/6J male mice (aged 8–12 weeks) were acclimatized to the behavioral testing suite at least 2 hours prior to the test. Mice were injected (intraperitoneally) with vehicle (5% glucose) or scopolamine (1.5 mg/kg) alone or in combination with xanomeline, GSK1034702, or TBPB 30 minutes prior to training. Mice were placed in the conditioning chamber (ANY-maze Fear Conditioning System; Stoelting, Dublin, Ireland); after a 2-minute adaptation period, they received three tone/foot shock pairings where the foot shock (unconditioned stimulus; 2 seconds; 0.4 mA) always co-terminated with a tone (conditioned stimulus; 2.8 kH; 85 dB; 30 seconds). The conditioned stimulus–unconditioned stimulus pairings were separated by 1-minute intervals. After the mice completed training, they remained in the conditioning chamber for 1 minute and were then returned to their home cages. The next day, the mice were placed back in the conditioning chamber, and time spent immobile was recorded for 3 minutes to assess context-dependent learning. Data were analyzed using ANY-maze software (Stoelting).

#### Mouse Pharmacokinetics.

Pharmacokinetic analyses were conducted as previously described (Witkin et al., 2017). Compounds were administered via intraperitoneal injection (in 5% glucose) 30 minutes prior to blood collection. Mice were anesthetized with 3% isoflurane (2 l/min), and blood was collected by cardiac puncture of the left ventricle. Blood was immediately transferred to EDTA tubes and centrifuged at 1000*g* for 10 minutes at 4°C; the supernatant was collected and frozen. Brains from each mouse were also dissected and snap-frozen on dry ice.

Brain samples were homogenized in three volumes of methanol/water [1:4 (v/v)] by weight. A 25-*μ*l aliquot of each study sample, calibration standard, and control sample was added to a 96-well plate and mixed with 180 *μ*l acetonitrile/methanol [1:1 (v/v)] containing internal standard. The samples were subsequently centrifuged, and the resulting supernatants were diluted 12.5-fold with methanol/water [1:1 (v/v)] prior to analyzing 10-*μ*l aliquots by liquid chromatography–tandem mass spectrometry as previously described (Bradley et al., 2017).

#### Equilibrium [^3^H]-NMS Binding.

Chinese hamster ovary (CHO) Flp-In cells expressing the wild-type M_1_ mAChR (*B*_max_ = 870 fmol/mg) or the M_1_ designer receptor exclusively activated by designer drug (DREADD) (*B*_max_ = 2400 fmol/mg) were plated at 7500 cells/well in clear 96-well plates and grown to confluence. Prior to the experiment, cells were washed with 100 *μ*l phosphate-buffered saline. Increasing concentrations of test compounds and an approximate equilibrium dissociation constant (*K*_D_) concentration of [^3^H]-NMS (*K*_D_ (nM) for [^3^H]-NMS binding to CHO Flp-In cells expressing wild-type M_1_ mAChR or M_1_ DREADD were 0.37 ± 0.10 and 18.70 ± 3.49 (*n* = 3) were incubated with cells overnight at room temperature in a final volume of 100 *μ*l binding buffer of the following composition: 110 mM NaCl, 5.4 mM KCl, 1.8 mM CaCl_2_, 1 mM MgSO_4_, 25 mM glucose, 20 mM HEPES, and 58 mM sucrose, pH 7.4. Binding was terminated by rapid aspiration followed by two washes with 200 *μ*l ice-cold 0.9% NaCl. Bound radioactivity was determined by liquid scintillation (Ultima Gold; PerkinElmer, Boston, MA) counting. Nonspecific binding was determined in the presence of 10 *μ*M atropine.

For competition binding experiments at M_1_, M_2_, M_3_, M_4_, and M_5_ mAChRs, CHO membranes were purchased from PerkinElmer. All experiments were performed in assay buffer of the following composition: 20 mM HEPES, 100 mM NaCl, and 10 mM MgCl_2_, pH 7.5, and used 10 *µ*g protein/well in a total assay volume of 1 ml using deep well blocks. CHO cell membranes overexpressing human mAChR M_1_–M_5_ subtypes were incubated with a concentration of [^3^H]-NMS that was close to the calculated *K*_D_ for each receptor (M_1_: 200 pM, *K*_D_ = 196 pM; M_2_: 700 pM, *K*_D_ = 769 pM; M_3_: 700 pM, *K*_D_ = 642 pM; M_4_: 200 pM, *K*_D_ 143 pM; M_5_: 400 pM, *K*_D_ = 410 pM), in the presence or absence of 11 different concentrations of compound. Nonspecific binding was determined in the presence of 10 *µ*M atropine. All assay incubations were initiated by the addition of membrane suspensions and deep well blocks were shaken for 5 minutes to ensure complete mixing. Incubation was then carried out for 2 hours at 21°C. Binding reactions were terminated by rapid filtration through GF/A filters (PerkinElmer) presoaked with 0.5% (w/v) polyethylenimine for 1 hour. Filters were then washed three times with 1 ml ice-cold assay buffer. Dried filters were counted with Meltilex A scintillant using a Trilux 1450 scintillation counter (PerkinElmer). The specific bound counts (in disintegrations per minute) were expressed as a percentage of the maximal binding observed in the absence of test compound (total) and nonspecific binding determined in the presence of 10 *µ*M atropine.

#### Kinetic [^3^H]-NMS Binding.

For determination of [^3^H]-NMS dissociation kinetics, membranes (5 *μ*g/tube) expressing the M_1_ mAChR were preincubated with [^3^H]-NMS for 1 hour at 37°C in binding buffer containing 100 mM NaCl, 10 mM MgCl_2_, and 20 mM HEPES, pH 7.4. Dissociation of the bound radioligand was initiated by the addition of atropine (10 *μ*M) alone or atropine (10 *μ*M) plus 100 *μ*M GSK1034702 added in a reverse time course protocol. Reactions were terminated by rapid filtration onto GF/B filter paper (Whatman, Maidstone, UK) and three washes with 3 ml ice-cold 0.9% NaCl using a Brandel harvester (M-24TI; Brandel, Fort Lauderdale, FL). Membrane bound radioactivity was determined by liquid scintillation (Ultima Gold; PerkinElmer) counting.

#### IP-One Accumulation Assay.

Stimulation of IP accumulation was determined using the Cisbio IP-One Gq assay kit per the manufacturer’s instructions. For agonist concentration-response curves, agonists (2× concentrated) were added to 384-well white ProxiPlates (PerkinElmer) in 7 *μ*l stimulation buffer. CHO Flp-In cells stably expressing the human M_1_ mAChR were grown to confluence in T75 cell culture flasks at 37°C. Cells were washed with warm phosphate-buffered saline and detached using phosphate-buffered saline with 0.1 M EDTA. Detached cells were centrifuged at 1000*g* and the cells were resuspended in stimulation buffer. Seven microliters of this cell suspension (1.43 × 10^6^ cells/ml) was added to each well, and cells were stimulated for 45 minutes at 37°C.

For functional interaction studies, CHO Flp-In cells stably expressing the human M_1_ mAChR were seeded at 5000 cells/well in 384-well white ProxiPlates. Experiments were conducted 48 hours later. Cells were washed once with 50 *μ*l phosphate-buffered saline and then incubated in F12 media containing phenoxybenzamine (where applicable) at 37°C for 30 minutes. Cells were washed with 50 *μ*l phosphate-buffered saline and incubated in stimulation buffer containing agonists in a final volume of 14 *μ*l for 45 minutes at 37°C.

All IP-One stimulations were terminated by the addition of 3 *μ*l/well IP_1_-d2 solution, followed by 3 *μ*l/well anti–IP_1_-cryptate solution and incubation for 1 hour at room temperature with shaking. Fluorescence emission at two different wavelengths (665 and 620 nm) was measured with a PHERAstar plate reader (BMG Labtech, Ortenberg, Germany).

#### ERK1/2 Phosphorylation.

Stimulation of phospho-ERK1/2 (Thr 202/Tyr 204) was determined using the Cisbio Phospho-ERK Cellular Assay Kit. CHO Flp-In cells stably expressing the human M_1_ receptor were seeded onto transparent 96-well plates at 20,000 cells/well and grown to confluence. Cells were serum starved overnight prior to the experiment. Prior to the stimulations, cells were washed with 100 *μ*l phosphate-buffered saline and then incubated in serum free F12 medium at 37°C. Cells were stimulated with ligands for 5 minutes at 37°C in a final volume of 200 *μ*l. The stimulations were terminated by rapid aspiration and addition of 50 *μ*l lysis buffer supplemented with blocking reagent per the manufacturer’s instructions, followed by gentle agitation at room temperature for 30 minutes. Subsequently, 16 *μ*l of this lysate was transferred to a 384-well white ProxiPlate (PerkinElmer) and incubated with 4 *μ*l premixed antibody solution for 2 hours at room temperature. Fluorescence emission at two different wavelengths (665 and 620 nm) was measured with a PHERAstar plate reader (BMG Labtech).

#### Native Tissue GTPɣ[^35^S] Binding Assays.

GTPɣ-[^35^S] ([^35^S]ɣ[guanosine-5′-O-(3-[^35^S]thio)triphosphate] binding in rat membranes was determined in triplicate using an antibody capture technique in 96-well plate format (DeLapp et al., 1999). Native rat membranes were prepared as follows. All procedures were performed at 4°C. Ten to fifteen milliliters of sucrose buffer (10 mM HEPES, 1 mM EGTA, 1 mM dithiothreitol, 10% sucrose, and one tablet/50 ml Complete Protease Inhibitor Cocktail, pH 7.4) was added to each tissue sample and homogenized for 10 strokes using an electric IKA RW20 homogenizer (800 rpm) (IKA, Staufen, Germany) with glass homogenizer. The homogenate was centrifuged at 1000*g* for 10 minutes at 4°C and the supernatant collected. The pellet was rehomogenized and centrifuged again as above and the supernatant pooled and centrifuged at 11,000*g* for 20 minutes at 4°C. The resulting pellet was suspended in 40 ml final storage buffer (10 mM HEPES, 1 mM EGTA, 1 mM MgCl_2_, and 1 mM dithiothreitol, pH 7.4) and centrifuged at 27,000*g* for 20 minutes at 4°C. The supernatant was removed and the final pellet was suspended in 2 ml final storage buffer. The protein concentration was measured using the Bradford method (Coomassie Plus, Bio-Rad protein assay kit; Bio-Rad, Hercules, CA) with bovine gamma globulin standards. Samples were aliquoted and stored at −80°C. Membrane aliquots (15 *µ*g/well) were then incubated with test compound and GTPɣ[^35^S] (500 pM/well) for 30 minutes. Labeled membranes were then solubilized with 0.27% Nonidet P-40 plus Gq*α* antibody (E17; Santa Cruz Biotechnology, Dallas, TX) at a final dilution of 1:200 and 1.25 mg/well anti-rabbit scintillation proximity beads. Plates were left to incubate for 3 hours and then centrifuged for 10 minutes at 2000 rpm. Plates were counted for 1 minute/well using a Wallac MicroBeta Trilux scintillation counter (PerkinElmer). All incubations took place at room temperature in GTP-binding assay buffer of the following composition: 20 mM HEPES, 100 mM NaCl, and 5 mM MgCl_2_, pH 7.5. Data were converted to the percentage of response compared with oxotremorine-M (100 *µ*M) or the percentage over basal and EC_50_ values were generated (four-parameter logistic curve) using GraphPad Prism 6 software (GraphPad Software Inc., La Jolla, CA).

#### [^3^H]-GSK1034702 Binding.

[^3^H]-GSK1034702 (specific activity 3.65 TBq/mmol) was synthesized by direct titration on Pd Black (performed by Quotient Bioresearch, Manchester, UK). All experiments were performed in assay buffer of the following composition: 20 mM HEPES, 100 mM NaCl, and 10 mM MgCl_2_, pH 7.5, and used 50 *µ*g protein/well in a total assay volume of 250 *μ*l.

CHO cell membranes overexpressing human M_1_ mAChR (PerkinElmer) were incubated with [^3^H]-GSK1034702 (20 nM) in the presence of 11 concentrations of test compound. All assay incubations were initiated by the addition of membrane suspensions. Incubation was then carried out for 4 hours at 21°C. Binding reactions were terminated by rapid filtration through GF/A filters (PerkinElmer) presoaked with 0.5% (w/v) polyethylenimine for 1 hour. Filters were then washed six times with 1 ml ice-cold assay buffer. Dried filters were counted using Meltilex A scintillant using a Trilux 1450 scintillation counter (PerkinElmer). The specific bound counts (in disintegrations per minute) were expressed as a percentage of the maximal binding observed in the absence of test compound (total) and nonspecific binding determined in the presence of 100 *µ*M nonradiolabeled GSK1034702.

#### Isolated Rat Atria and Ileum Contraction Experiments.

Adult Wistar rats were humanely euthanized and the left atria and the ileum were placed in an organ bath containing oxygenated Tyrode solution. Measurement of negative inotropic responses in atria or contraction of ileum was performed as described by Lambrecht et al. (1989). For atrial responses, ligands were administered for 5 minutes at 32°C in McEwen’s buffer (pH 7.4) in a bath volume of 10 ml. Agonist responses were measured as negative inotropy relative to 1 *μ*M methacholine, and antagonism was measured as inhibition of methacholine (1 *μ*M)–induced negative ionotropic response. For rat ileum experiments, ligands were administered for 5 minutes at 32°C in Krebs’ buffer (pH 7.4) in a bath volume of 10 ml. Contraction of ileum as a percentage of methacholine-induced contraction was measured, and antagonistic effects were measured by inhibition of methacholine-induced responses.

#### Data Analysis.

Inhibition binding data were curve-fit using GraphPad Prism 6 to derive the potency (IC_50_) of the test compound. The equilibrium dissociation constant (*K*_I_) of the test compound was then calculated with the Cheng–Prusoff equation: *K*_I_ = IC_50_/[1 + ([L]/*K*_D_)] using the *K*_D_ value derived separately from saturation binding studies.

Functional concentration-response curves were fitted according to a four-parameter logistic equation (to determine minimum and maximum asymptotes, LogEC_50_, and slope; GraphPad Prism 6). For ACh curves in the presence of multiple concentrations of GSK1034702 or TBPB (after phenoxybenzamine treatment), the following form of the Gaddum and Schild equations was applied globally to the datasets:
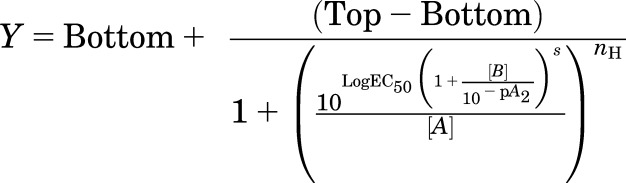
where top represents the maximal asymptote of the curves, bottom represents the minimum asymptote of the curves, LogEC_50_ represents the logarithm of the ACh EC_50_ in the absence of GSK1034702 or TBPB, [*A*] represents the concentration of ACh, [*B*] represents the concentration of GSK1034702 or TBPB, *n*_H_ represents the Hill slope of the agonist curve, *s* represents the Schild slope for the antagonist, and p*A*_2_ represents the negative logarithm of the concentration of antagonist that shifts the agonist EC_50_ by a factor of 2. In the absence of antagonist ([*B*] = 0), this equation becomes the standard four-parameter logistic equation for fitting agonist concentration-response data.

[^3^H]-NMS binding interaction studies with benzyl quinolone carboxylic acid (BQCA) were fitted to an allosteric ternary complex model (Leach et al., 2010):
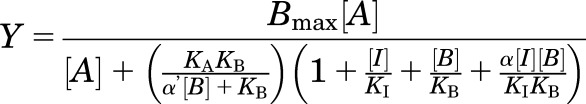
where *B*_max_ represents the total number of receptors; [*A*], [*B*], and [*I*] are concentrations of radioligand, allosteric modulator, and orthosteric ligand, respectively; and *K*_A_, *K*_B_, and *K*_I_ represent equilibrium dissociation constants of radioligand, allosteric modulator, and orthosteric ligand, respectively. *α*′ and *α* are the binding cooperativities between the allosteric modulator and radioligand and the allosteric modulator and the orthosteric ligand, respectively. An *α* value of >1 denotes positive cooperativity, a value of <1 denotes negative cooperativity, and a value of 1 denotes neutral cooperativity of binding.

To assess agonist bias, the same concentration-response curves were analyzed according to a modified form of the operational model of agonism, recast to directly yield a transduction ratio (Log[*τ*/*K*_A_]; van der Westhuizen et al., 2014):
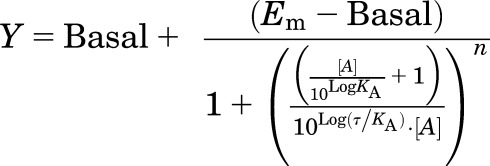
where basal represents the response in the absence of agonist, *E*_m_ represents the maximal response of the assay system, *K*_A_ represents the equilibrium dissociation constant of the agonist, [*A*] represents the concentration of agonist, *τ* is an index of the coupling efficiency (or efficacy) of the agonist, and *n* is the slope of the transducer function linking agonist occupancy to response. For the analysis, all families of agonist curves at each pathway were globally fitted to the model with the parameters, basal, *E*_m_, and *n* shared between all agonists. For full agonists, the Log*K*_A_ was constrained to a value of zero, whereas for partial agonists this was directly estimated by the curve fitting procedure; the Log(*τ/K*_A_) parameter was estimated as a unique measure of activity for each agonist. Agonist bias factors (10^ΔΔLog[*τ*/K_A_]) were calculated as described in van der Westhuizen et al. (2014)).

## Results

### 

#### GSK1034702, TBPB, and Xanomeline Reverse Scopolamine-Induced Deficits in Fear Conditioning.

**Fig. 1.**
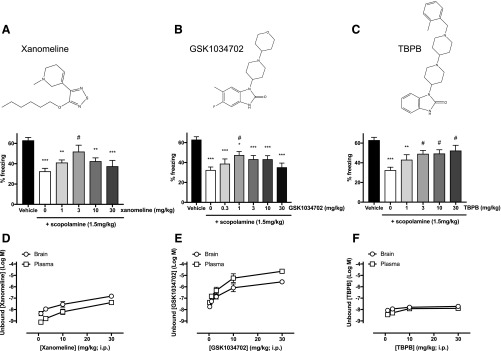
(A–C) Effects of xanomeline (1, 3, 10, and 30 mg/kg) (A), GSK1034702 (0.3, 1, 3, 10, and 30 mg/kg) (B), and TBPB (1, 3, 10, and 30 mg/kg) (C) on scopolamine (1.5 mg/kg)–induced impairments in contextual fear conditioning (inset chemical structures of compounds). Data are expressed as the means ± S.E.M. of eight or more mice per group. Data were analyzed using a one-way analysis of variance with the Tukey multiple comparison test. *P < 0.05; ** *P* < 0.01; ***P < 0.001 vs. vehicle alone; ^#^*P* < 0.05 vs. 1.5 mg/kg scopolamine. (D–F) Unbound concentrations (Log M) of xanomeline (D), GSK1034702 (E), and TBPB (F) measured in plasma or brain samples 30 minutes after intraperitoneal injection with increasing concentrations of respective compound. Data are expressed as the means ± S.E.M. of three mice per concentration.

It is well established that muscarinic receptor agonists and PAMs can reverse deficits in learning and memory induced by the administration of a broad-spectrum muscarinic antagonist such as scopolamine (Young et al., 1995; Ma et al., 2009). Here, doses of scopolamine above 1 mg/kg administered to mice 30 minutes prior to fear conditioning training were sufficient to induce a significant reduction in contextual fear conditioning learning and memory (Supplemental Fig. 1). The effects of muscarinic receptor agonists on this deficit were tested by the coadministration of scopolamine (1.5 mg/kg) with escalating intraperitoneal doses of xanomeline (Fig. 1A), GSK1034702 (Fig. 1B), or TBPB (Fig. 1C). All three agents significantly improved learning and memory compared with vehicle controls (5% glucose solution in double distilled water) (*P* < 0.05 vs. administration of 1.5 mg/kg scopolamine alone; one-way analysis of variance with the Tukey multiple comparisons test). Free brain concentrations of xanomeline and GSK1034702 determined 30 minutes after administration were seen to increase linearly with escalating doses (Fig. 1, D and E). In contrast, the effects of these compounds on learning and memory were bell shaped, with lower doses improving learning and memory and high doses showing reduced effect (Fig. 1, A and B). This bell-shaped response is characteristic of procognitive agents. Interestingly, brain exposure of TBPB could not be increased beyond that observed at 10 mg/kg, remaining relatively low even after intraperitoneal injection of higher doses (Fig. 1F). This resulted in TBPB effects on learning and memory being similar at both low and high-administered doses with no evidence of a bell-shaped dose response (Fig. 1C).

#### GSK1034702 and TBPB Interact Competitively with [^3^H]-NMS at M_1_ mAChRs.

**Fig. 2.**
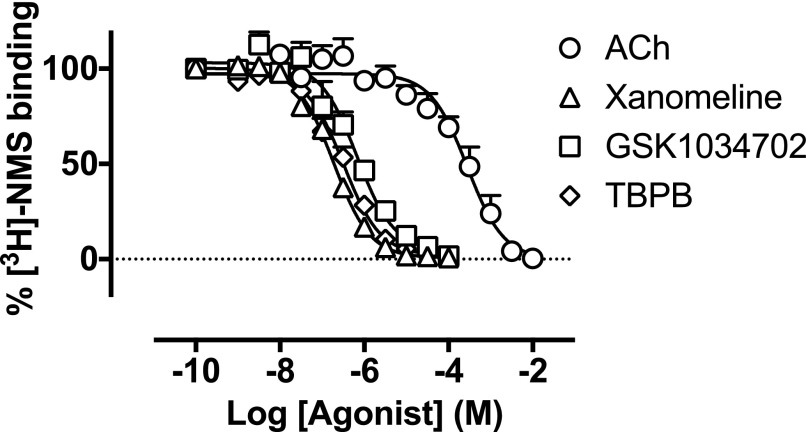
Displacement of [^3^H]-NMS binding by ACh, xanomeline, GSK1034702, or TBPB at the human M_1_ mAChR expressed in CHO Flp-In cell monolayers. Experiments were performed against a *K*_D_ concentration of [^3^H]-NMS. Nonspecific binding was determined by the addition of 10 *μ*M atropine. Data are expressed as the means ± S.E.M. of three to five independent experiments performed in duplicate.

GSK1034702 and TBPB have previously been described as allosteric agonists of the M_1_ mAChR (Jones et al., 2008; Budzik et al., 2010; Nathan et al., 2013). To test this assertion, [^3^H]-NMS binding studies were conducted on monolayers of CHO Flp-In cells expressing the human M_1_ mAChR to determine the nature of their interaction at the receptor. Both GSK1034702 and TBPB fully inhibited binding of [^3^H]-NMS (0.5 nM) to M_1_ mAChRs, with estimated p*K*_i_ values of 6.5 ± 0.2 and 6.8 ± 0.1, respectively, and in a similar manner to the orthosteric agonist, xanomeline (Fig. 2; Table 1). These data suggest that GSK1034702, in contrast to the allosteric mechanism of action previously reported, binds instead in a competitive manner consistent with interaction at the orthosteric site of the M_1_ mAChR.

**TABLE 1:** Affinity estimates for the competition between [^3^H]-NMS and ACh, xanomeline, GSK1034702, or TBPB at the M_1_ mAChR Values stated are the negative logarithms of the equilibrium dissociation constant (p*K*_i_). Data are calculated from the means ± S.E.M. of three to five independent experiments performed in duplicate.

Compound	p*K*_i_	*n*
ACh	4.1 ± 0.3	5
Xanomeline	7.0 ± 0.1	4
GSK1034702	6.5 ± 0.2	3
TBPB	6.8 ± 0.1	3

#### GSK1034702 and TBPB Do Not Alter [^3^H]-NMS Dissociation Kinetics.

**Fig. 3.**
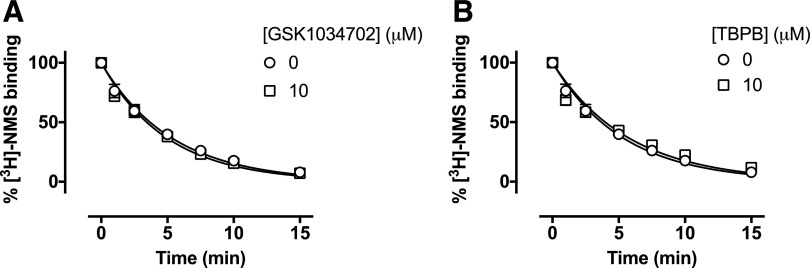
(A and B) Dissociation of [^3^H]-NMS with atropine in the absence or presence of GSK1034702 (A) or TBPB (B) in membranes expressing the human M_1_ receptor. Membranes were incubated with a *K*_D_ concentration of [^3^H]-NMS for 60 minutes at 37°C, followed by dissociation of bound radioligand with atropine (10 *μ*M) alone or in the presence of GSK1034702 (10 *μ*M) or TBPB (10 *μ*M). Data shown are the mean of three independent experiments performed in duplicate.

As allosteric ligands with high negative cooperativity can still fully inhibit orthosteric ligand binding, kinetic binding experiments were performed to probe any allosteric interactions of GSK1034702 or TBPB with the M_1_ mAChR. Membranes of CHO Flp-In cells expressing the human M_1_ mAChR were pre-equilibrated with [^3^H]-NMS, and bound radioligand dissociated from the receptor with atropine (10 *μ*M) with a rate constant of *k*_off_ = 0.188 ± 0.009 minute^−1^. The presence of either GSK1034702 (10 *μ*M) or TBPB (10 *μ*M) had no effect on the [^3^H]-NMS dissociation rate (Fig. 3). These data further argue against an allosteric mode of action of GSK1034702 at M_1_ mAChRs as previously reported (Nathan et al., 2013).

#### Receptor Alkylation Studies Establish Orthosteric Binding of GSK1034702.

**Fig. 4.**
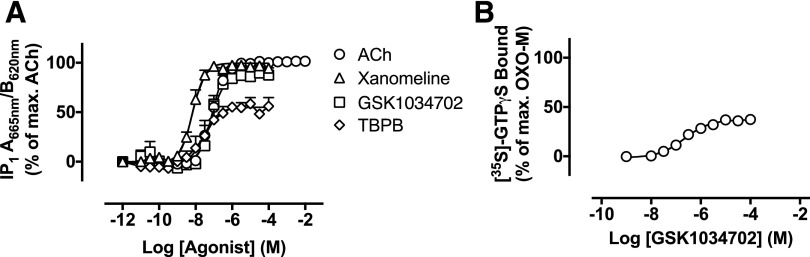
(A) IP accumulation elicited by ACh, xanomeline, GSK1034702, or TBPB via the human M_1_ receptor expressed in CHO Flp-In cells. Data are expressed as the means ± S.E.M. of 3–10 independent experiments performed in duplicate. (B) [^35^S]-GTP*γ*S binding to rat frontal cortex membranes. Data are the percentage of the maximal [^35^S]-GTP*γ*S binding stimulated by oxotremorine-M (mean pEC_50_ = 6.68 ± 0.13, *n* = 3). OXO-M, oxotremorine-M.

GSK1034702 activity in IP_1_ accumulation assays was compared with ACh, xanomeline, and TBPB (Fig. 4A; Table 2). GSK1034702 stimulated robust increases in IP_1_ accumulation, reaching approximately 90% of the maximal response elicited by ACh, with nanomolar potency (pEC_50_ = 7.1 ± 0.1; Fig. 4A). TBPB behaved as a partial agonist with a modest increase in potency relative to GSK1034702 (pEC_50_ = 7.6 ± 0.2). In membranes prepared from the rat cortex, GSK1034702 is a partial agonist with respect to G*α*_q_ protein coupling, stimulating approximately 37% of the maximum [^35^S]-GTP*γ*S G*α*_q_ binding elicited by the full agonist oxotremorine-M (pEC_50_ = 6.7 ± 0.1; Fig. 4B).

**TABLE 2:** Maximum agonist effect and potency of ACh, xanomeline, GSK1034702, and TBPB at stimulating IP_1_ accumulation in CHO Flp-In human M_1_ cells Data are expressed as the means ± S.E.M. of 3–10 independent experiments performed in duplicate.

Compound	IP_1_		
	*E*_max_	pEC_50_	*n*
ACh	100	7.1 ± 0.1	10
Xanomeline	98.0 ± 1.5	8.2 ± 0.1	3
GSK1034702	90.1 ± 2.9	7.1 ± 0.1	3
TBPB	55.2 ± 3.1	7.6 ± 0.2	4

To verify the mechanism of action, we performed receptor alkylation experiments with the orthosteric site covalent binder, phenoxybenzamine, to deplete the level of available and functional muscarinic receptors. Phenoxybenzamine, at a concentration of 3 *μ*M (for 30 minutes), reduced the functional human M_1_ mAChR population in CHO Flp-In cells by approximately 80%, to an expression level where GSK1034702 had no agonist effect in an IP_1_ accumulation assay but where ACh still yielded a response (Supplemental Fig. 2, A and B). Under these conditions, establishing whether GSK1034702 and TBPB acted as competitive antagonists with respect to ACh would verify the interaction of these compounds with the orthosteric site.

**Fig. 5.**
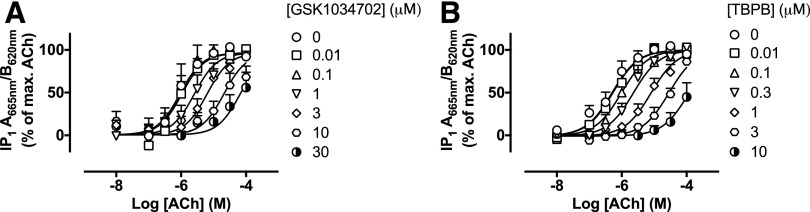
(A and B) GSK1034702 (A) or TBPB (B) antagonism of ACh-stimulated IP accumulation in CHO Flp-In cells expressing the human M_1_ receptor. Cells were incubated with 3 *μ*M phenoxybenzamine to irreversibly reduce receptor expression prior to the addition of GSK1034702 or TBPB. Data are expressed as the means ± S.E.M. of three independent experiments performed in duplicate.

In phenoxybenzamine-treated cells, GSK1034702 caused a concentration-dependent, parallel rightward shift in ACh-stimulated IP_1_ accumulation (Fig. 5A) consistent with a competitive antagonist. This effect was similar to that of TBPB (Fig. 5B), which was reported previously to act as a competitive antagonist in a similar preparation (Keov et al., 2013). Analysis of these data using a modified form of the Gaddum and Schild equations yielded Schild slopes approximating to unity and p*A*_2_ values of 6.2 ± 0.2 and 7.0 ± 0.1 for GSK1034702 and TBPB antagonism of ACh-stimulated responses, respectively (Table 3).

**TABLE 3:** Potency estimates of the antagonism of ACh-stimulated IP_1_ accumulation by GSK1034702 or TBPB in CHO Flp-In cells expressing the human M_1_ mAChR Data are the means ± S.E.M. of three independent experiments performed in duplicate.

Parameter	GSK1034702	TBPB	*n*
p*A*_2_	6.2 ± 0.2	7.0 ± 0.1	3
Schild slope	1.1 ± 0.1	1.2 ± 0.1	3

#### The Prototypical PAM, BQCA, Potentiates ACh, But Not GSK1034702 Affinity.

**Fig. 6.**
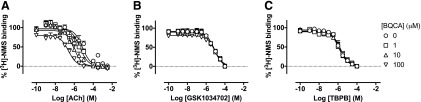
(A–C) Displacement of [^3^H]-NMS binding by ACh (A), GSK1034702 (B), or TBPB (C) in the presence of increasing concentrations of BQCA at the human M_1_ mAChR expressed in CHO Flp-In cell monolayers. Experiments were performed against a *K*_D_ concentration of [^3^H]-NMS. Nonspecific binding was determined by the addition of 10 *μ*M atropine. Data are expressed as the means ± S.E.M. of three independent experiments performed in duplicate.

Having established that GSK1034702 interacts competitively with ACh at the orthosteric site, radioligand binding experiments were designed to establish whether the mode of GSK1034702 binding at the orthosteric pocket was equivalent to ACh. In these studies, the potentiation of orthosteric agonist binding by a PAM was used to probe the nature of GSK1034702 and TBPB binding. BQCA, a PAM selective for the M_1_ mAChR, has previously been shown to potentiate ACh affinity by approximately 100-fold (Ma et al., 2009; Butcher et al., 2016). Consistent with these previous studies, we show here that BQCA potentiates the ACh-mediated displacement of [^3^H]-NMS, thereby demonstrating positive cooperativity for ACh binding of approximately 35-fold, consistent with previous reports of modulation according to a two-state model (Canals et al., 2012; Fig. 6A). Such actions would predict a similar (if less substantial) effect on GSK1034702 or TBPB. However, the displacement of [^3^H]-NMS by GSK1034702 (Fig. 6B) or TBPB (Fig. 6C) was not modulated by BQCA. These data demonstrate the probe dependency of BQCA and indicate that either: 1) there is neutral cooperativity between BQCA and GSK1034702/TBPB, or 2) that the binding site of GSK1034702 or TBPB simultaneously overlaps with those of both ACh and BQCA.

#### [^3^H]-GSK1034702 Binding Studies Further Confirm Novel Orthosteric Binding Pose.

**Fig. 7.**
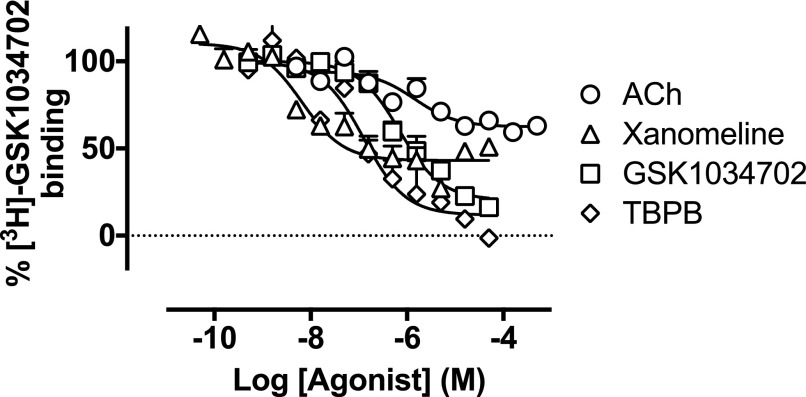
Displacement of [^3^H]-GSK1034702 binding by ACh, xanomeline, GSK1034702, or TBPB at membranes expressing the human M_1_ mAChR. Experiments were performed against 20 nM [^3^H]-GSK1034702 and nonspecific binding was determined in the presence of 100 *μ*M nonradiolabeled GSK1034702. Data are expressed as the means ± S.E.M. of three independent experiments performed in duplicate.

To further characterize the binding site of GSK1034702 at the M_1_ mAChR, we generated a radiolabeled version of the GSK1034702 compound ([^3^H]-GSK1034702) and conducted binding interaction experiments in membranes expressing the human M_1_ mAChR. [^3^H]-GSK1034702 bound in a monophasic manner with moderate affinity (*K*_D_ = 550 nM; *B*_max_ = 2.6 pmol/mg protein) (Supplemental Fig. 3, A–C). Membranes were incubated with 20 nM [^3^H]-GSK1034702 in the absence and presence of increasing concentrations of unlabeled GSK1034702, TBPB, ACh, and xanomeline (Fig. 7). GSK1034702 and TBPB fully displaced specific [^3^H]-GSK1034702 binding to the M_1_ mAChR in a monophasic manner, whereas ACh and xanomeline only partially displaced [^3^H]-GSK1034702 binding. These data indicate either an allosteric interaction between GSK1034702 and ACh/xanomeline or a bitopic mechanism consistent with GSK1034702 spanning a binding pocket at the M_1_ mAChR partially shared with that of ACh.

#### DREADD Pharmacology Confirms an Atypical Mechanism of Action of GSK1034702.

**Fig. 8.**
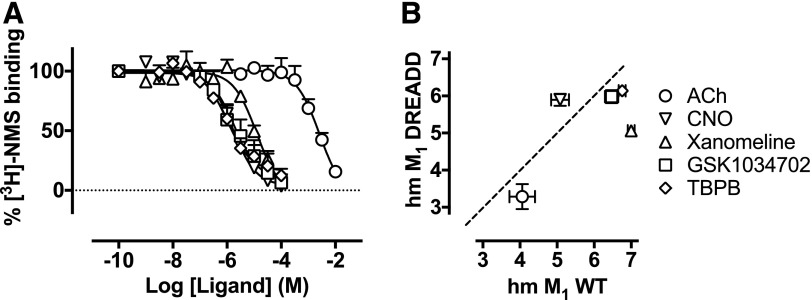
(A) Displacement of [^3^H]-NMS binding by ACh, CNO, xanomeline, GSK1034702, or TBPB at the humanized M_1_ DREADD expressed in CHO Flp-In cell monolayers. Experiments were performed against a *K*_D_ concentration of [^3^H]-NMS. Nonspecific binding was determined by the addition of 10 *μ*M atropine. Data are expressed as the means ± S.E.M. of three to five independent experiments performed in duplicate. (B) Comparison of p*K*_i_ values for each of the compounds used at the wild-type M_1_ mAChR or the mutant M_1_ DREADD. CNO, clozapine-*N*-oxide; WT, wild type.

**Fig. 9.**
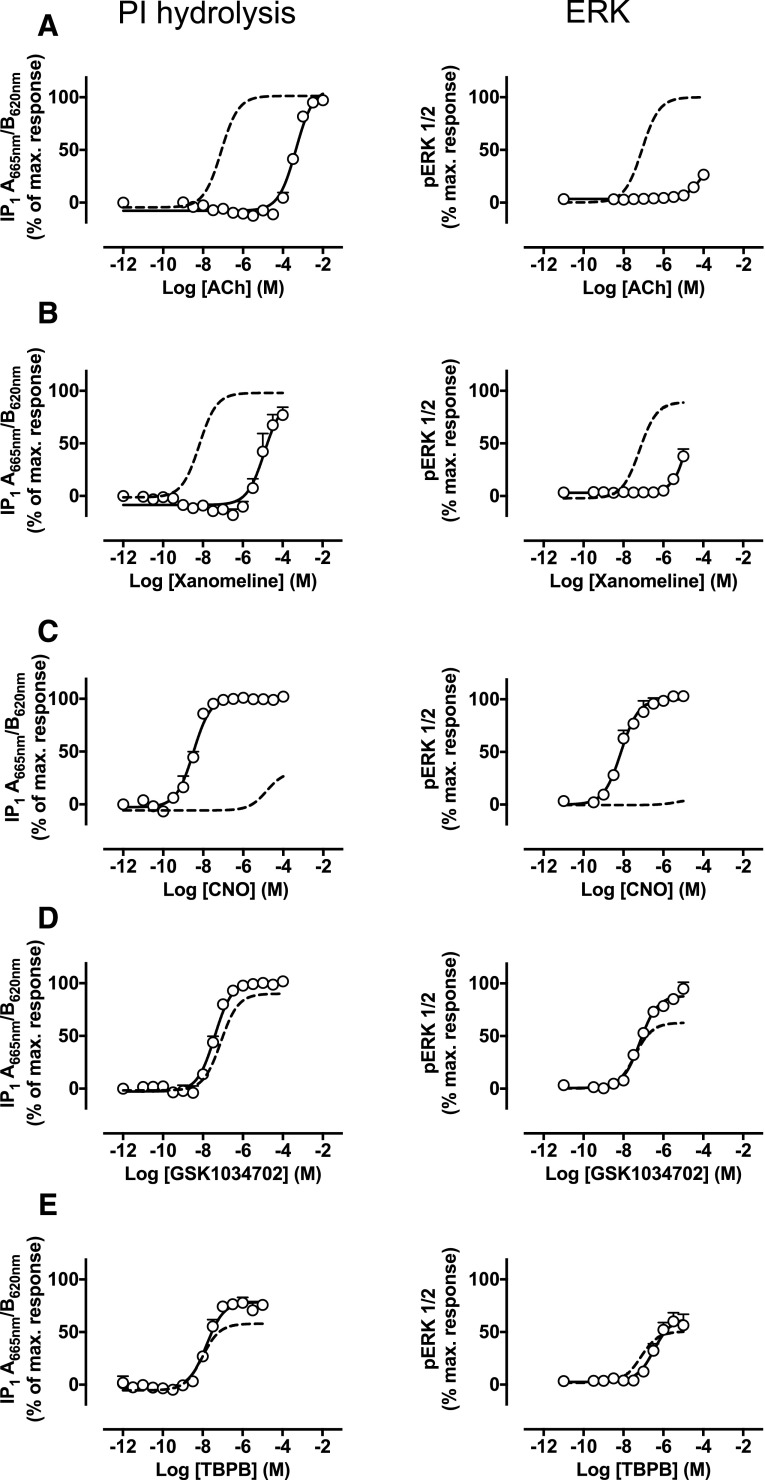
(A–E) IP accumulation (left) or ERK1/2 phosphorylation (right) elicited by ACh (A), xanomeline (B), CNO (C), GSK1034702 (D), or TBPB (E) via the humanized M_1_ DREADD expressed in CHO Flp-In cells. The dashed curve represents the response of the ligand at the wild-type M_1_ mAChR. Data are expressed as the means ± S.E.M. of three to four independent experiments performed in duplicate. CNO, clozapine-*N*-oxide; PI, phosphoinositide.

**Fig. 10.**
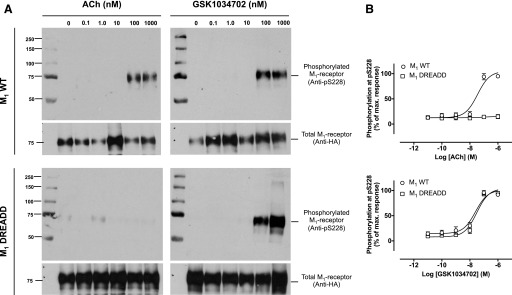
(A) Phosphorylation at serine 228 elicited by ACh (left) or GSK1034702 (right) in CHO Flp-In cells expressing either M_1_ WT (top) or M_1_ DREADD (bottom). (B) Mean densitometric data showing phosphorylation at serine 228 as a percentage of the maximal response. Data were normalized to the total receptor expression, assessed using an HA antibody. Data are expressed as the means ± S.E.M. of three independent experiments. HA, hemagglutinin; WT, wild type.

By introducing point mutations (Y106C and A196G) into the orthosteric binding pocket of the M_1_ mAChR, an M_1_ DREADD mutant is created (Abdul-Ridha et al., 2013) that displays reduced responsiveness to ACh but instead is activated by clozapine-*N*-oxide, a ligand that shows little activity at the wild-type M_1_ mAChR. We investigated the ability of GSK1034702 to interact with the M_1_ DREADD by conducting [^3^H]-NMS binding, functional IP_1_ accumulation, and ERK1/2 phosphorylation studies in CHO Flp-In cells expressing the humanized M_1_ DREADD (Figs. 8 and 9; Supplemental Tables 1 and 2). The affinity of GSK1034702 for the M_1_ DREADD was not significantly different from the affinity for binding at the wild-type M_1_ mAChR (p*K*_i_ = 6.5 ± 0.2 and 6.0 ± 0.2 for the wild type and DREADD, respectively; Fig. 8). In addition, as reported previously (Armbruster et al., 2007; Abdul-Ridha et al., 2013), M_1_ mAChR orthosteric agonists, ACh and xanomeline, showed a significant reduction in potency at the M_1_ DREADD (Fig. 9). In contrast, GSK1034702 activated the M_1_ DREADD with comparable potency and efficacy compared with its activity at the wild-type receptor. Similar results were obtained with TBPB, confirming previous observations for this compound (Abdul-Ridha et al., 2014) (Fig. 9, D and E). Furthermore, we assessed the ability of ACh and GSK1034702 to stimulate phosphorylation of the M_1_ mAChR at serine 228 using a phosphorylation-specific antibody (Butcher et al., 2016). Both ACh and GSK1034702 stimulated a concentration-dependent increase in pSer228 immunoreactivity at the wild-type receptor (Fig. 10). The potency of GSK1034702 to stimulate phosphorylation at serine 228 was unchanged at the M_1_ DREADD, whereas ACh failed to stimulate a response. These data support the notion that GSK1034702 has a distinct binding mode at the orthosteric site from that of ACh.

#### GSK1034702 and TBPB Are Differentially Biased Agonists at M_1_ mAChR.

Convergent evidence from radioligand binding and functional studies (*vide supra* and Supplemental Fig. 4; Supplemental Table 3) suggests that GSK1034702 and TBPB bind to the orthosteric site of the M_1_ mAChR, but with an orientation or pose that distinguishes them from ACh and xanomeline. To further interrogate their pharmacology, we assessed their ability to engender biased signaling by application of an operational model of agonism to the concentration-response curves of either xanomeline, GSK1034702, or TBPB in both IP_1_ and ERK1/2 phosphorylation assays (Evans et al., 2011; Keov et al., 2011; Kenakin et al., 2012) (Table 4). These analyses generated transduction coefficient values for each of these agonists at the two different pathways and allowed us to calculate the bias factor between IP_1_ and ERK1/2 phosphorylation, revealing significant differences between ACh and xanomeline/TBPB (*P* < 0.001; one-way analysis of variance), with the latter displaying bias toward IP_1_ responses (bias factor IP_1_ − ERK1/2 phosphorylation = 1.1 and 1.4 for xanomeline and TBPB, respectively). However, no significant differences were revealed for GSK1034702 and ACh, suggesting that GSK1034702 and TBPB, despite apparently similar binding modes, engender differential signaling from the receptor.

**TABLE 4:** Transduction coefficients [Log10(*τ*/KA)], normalized (reference ligand ACh) transduction coefficients [ΔLog10(*τ*/K_A_)], and bias factors [ΔΔLog10(*τ*/KA)] for IP_1_ accumulation and phosphorylation of ERK1/2 at the wild-type M_1_ mAChR

Compound	IP_1_ Accumulation		ERK1/2 Phosphorylation		Log Bias Factor IP_1_ – ERK1/2
	Log10(*τ*/K_A_)	ΔLog10(*τ*/K_A_)	Log10(*τ*/K_A_)	ΔLog10(*τ*/K_A_)	ΔΔLog10(*τ*/K_A_)
ACh	7.10 ± 0.1	0.0 ± 0.1	7.1 ± 0.0	0.0 ± 0.0	0.0 ± 0.1
Xanomeline	8.2 ± 0.1	1.1 ± 0.1	7.1 ± 0.3	0.0 ± 0.3	1.1 ± 0.1
GSK1034702	7.4 ± 0.2	0.3 ± 0.2	7.2 ± 0.1	0.1 ± 0.1	0.2 ± 0.2
TBPB	8.2 ± 0.2	1.1 ± 0.2	6.9 ± 0.4	−0.3 ± 0.4	1.4 ± 0.2

#### GSK1034702 Shows a Lack of Selectivity for M_1_ mAChRs.

**Fig. 11.**
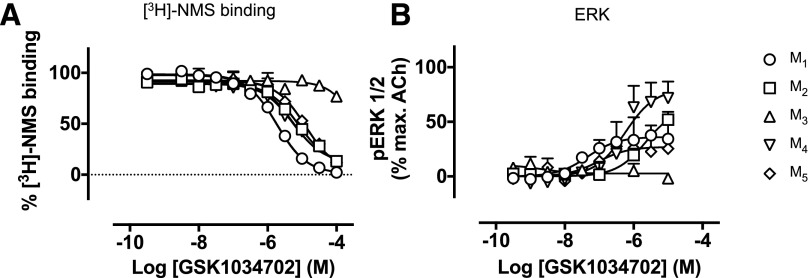
(A) Displacement of [^3^H]-NMS binding by GSK1034702 in CHO membranes expressing M_1_, M_2_, M_3_, M_4_, or M_5_ mAChRs. Experiments were performed against a *K*_D_ concentration of [^3^H]-NMS. Nonspecific binding was determined by the addition of 10 *μ*M atropine. Data are expressed as the means ± S.E.M. of three independent experiments. (B) ERK1/2 phosphorylation elicited by GSK1034702 at the M_1_, M_2_, M_3_, M_4_, or M_5_ mAChR expressed in CHO cells. Data are expressed as a percentage of the maximum response stimulated by ACh and are the means ± S.E.M. of three experiments performed in duplicate.

**Fig. 12.**
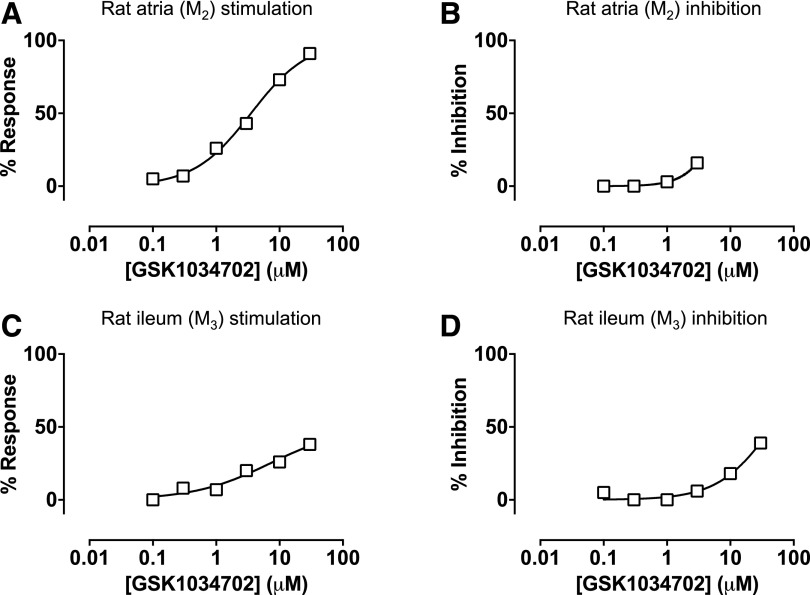
(A–D) Assessment of the effects of GSK1034703 activity at M_2_ (A and B) and M_3_ (C and D) mAChRs in ex vivo tissue preparations. The ability of GSK1034702 to stimulate negative inotropy in rat atria (A) or inhibit methacholine (1 *μ*M)–induced responses (B) was assessed. Activity of GSK1034702 at the M_3_ mAChR was assessed by measurement of rat ileum contraction relative to methacholine responses (C) or inhibition of methacholine-induced contraction (D). Data shown are a single experiment.

We evaluated the ability of GSK1034702 to bind to other mAChR subtypes by conducting equilibrium-binding studies on membranes expressing the M_1_, M_2_, M_3_, M_4_, or M_5_ mAChR, and we found that GSK1034702 could inhibit [^3^H]-NMS binding at all muscarinic receptor subtypes, albeit with much lower affinity for the M_3_ mAChR (Fig. 11A; Table 5). We further assessed the functional activity of GSK1034702 at the M_2_, M_3_, M_4_, and M_5_ mAChRs in the ERK1/2 phosphorylation assay. GSK1034702 exhibited partial agonist activity at M_2_, M_4_, and M_5_ mAChRs but was devoid of activity at the M_3_ mAChR in this assay (Fig. 11B). Finally, we investigated the ability of GSK1034702 to stimulate negative inotropic responses in isolated rat atria (Fig. 12, A and B) or contraction of rat ileum (Fig. 12, C and D), indicative of activity at M_2_ and M_3_ mAChRs, respectively. GSK1034702 elicited a robust response in the rat atria, reaching a maximal response equivalent to that of methacholine, with micromolar potency (Fig. 12A). Furthermore, GSK1034702 could inhibit methacholine-induced responses with an IC_50_ of 8 *μ*M (Fig. 12B). In the rat ileum, GSK1034702 stimulated approximately 50% of the maximal methacholine-induced contraction, with an EC_50_ of 7 *μ*M (Fig. 12C), and inhibited methacholine-induced contraction with an IC_50_ of 46 *μ*M (Fig. 12D).

**TABLE 5:** Negative logarithms of the equilibrium dissociation constant (p*K*_i_) of GSK1034702 binding to M_1_–M_5_ mAChRs Data are calculated from the means ± S.E.M. of three independent experiments performed in duplicate.

mAChR subtype	p*K*_i_	*n*
M_1_	6.0 ± 0.1	3
M_2_	5.4 ± 0.1	3
M_3_	n.d.	3
M_4_	5.7 ± 0.1	3
M_5_	5.2 ± 0.1	3

n.d., not determined.

## Discussion

The development of the selective M_1_ mAChR allosteric agonist GSK1034702 provided the opportunity to test the hypothesis that allosteric M_1_ mAChR drugs might provide a clinical advantage over orthosteric M_1_ mAChR agents due to increased selectivity while yielding fewer side effects. In the nicotine abstinence model of cognitive dysfunction, GSK1034702 significantly improved immediate memory recall but also induced adverse responses consistent with activation of other muscarinic receptor subtypes (Nathan et al., 2013). At first glance, these data might suggest that allosteric M_1_ mAChR drugs offer little or no safety benefit compared with previous investigational agents targeting mAChRs. However, here we provide direct pharmacological evidence that GSK1034702 is not a pure allosteric agonist as previously reported, but rather interacts with the orthosteric binding site and broadly mimics the pharmacology of the known bitopic ligand, TBPB. Based on radioligand binding and functional studies, coupled with the structural similarity between GSK1034702 and TBPB, we conclude that GSK1034702 likely interacts concomitantly with both allosteric and orthosteric sites on the M_1_ mAChR in a bitopic manner.

The conclusion that GSK1034702 interacts with the orthosteric site is primarily based on the full inhibition of [^3^H]-NMS binding and a lack of cooperative effects on [^3^H]-NMS, features consistent with an orthosteric rather than prototypical allosteric mechanism. In functional assays after receptor alkylation (to diminish its agonist response), GSK1034702 causes a nonsaturable, concentration-dependent parallel rightward shift in the ACh-mediated IP response, further confirming an orthosteric mode of action.

That GSK1034702 might also interact with a site distinct from the orthosteric site was indicated most clearly in functional assays at the M_1_ mAChR DREADD and by characterizing the binding of [^3^H]-GSK1034702 to the wild-type receptor. The M_1_ mAChR DREADD contains mutations at key residues within the orthosteric binding pocket, which yields a receptor that is poorly responsive to the cognate ligand, ACh, but instead is activated by an otherwise inert chemical ligand, clozapine-*n*-oxide (Armbruster et al., 2007; Roth, 2016). As predicted from previous reports (Abdul-Ridha et al., 2014), the potencies of orthosteric ligands ACh and xanomeline in IP signaling, ERK1/2 phosphorylation, and M_1_ receptor phosphorylation were significantly reduced at the M_1_ DREADD. However, the potency and efficacy of GSK1034702 and TBPB was unaffected by the DREADD mutations, suggesting that GSK1034702, like TBPB, is able to activate the M_1_ mAChR with a binding mode that is subtly distinct from that of prototypical orthosteric ligands. In support of this conclusion, GSK1034702 and TBPB fully inhibit the binding of [^3^H]-GSK1034702 to the M_1_ mAChR, whereas the prototypical orthosteric agonists, ACh and xanomeline, only partially inhibit its binding. This indicates that GSK1034702 can still bind to the M_1_ mAChR when ACh or xanomeline occupy the orthosteric site, suggesting that it can interact with the receptor via an allosteric binding site. The display of apparently both orthosteric (competitive) and allosteric behaviors depending on test system is typical of bitopic ligands that are able to “flip-flop” between binding poses (Valant et al., 2012).

Greater clarity around the receptor mechanism of action of GSK1034702, revealed here, has implications for drug design aimed at the treatment of AD. We have previously demonstrated that the learning and memory deficit observed in murine prion disease is due to a loss of cholinergic signaling in the hippocampus and as such, this model replicates one of the key pathologic hallmarks associated with human AD (Bradley et al., 2017). In the prion model, we found that both M_1_ mAChR orthosteric agonists and allosteric modulators completely rescue the learning and memory deficit observed in prion disease. However, we also found that the orthosteric ligand, xanomeline, gave adverse responses consistent with the activation of other muscarinic receptor subtypes, whereas the PAM, BQCA, gave no detectable adverse responses at doses that rescued learning and memory (Bradley et al., 2017). Although these results together with other studies on the cognitive responses of M_1_ mAChR allosteric modulators (Ma et al., 2009; Lange et al., 2015; Puri et al., 2015; Vardigan et al., 2015) support the potential clinical benefit of this class of ligand, it is also clear that allosteric modulators that show direct agonism in addition to cooperativity in rodent models result in adverse effects (Alt et al., 2016; Davoren et al., 2016, 2017). Hence, we conclude here that to avoid adverse effects, clinical candidates targeting the M_1_ mAChR in AD would require the following properties: 1) high levels of receptor subtype selectivity as would be seen with an allosteric modulator and 2) low levels of intrinsic agonist activity.

## Abbreviations

ACh: acetylcholine
AD: Alzheimer disease
BQCA: benzyl quinolone carboxylic acid
CHO: Chinese hamster ovary
DREADD: designer receptor exclusively activated by designer drug
ERK: extracellular signal–regulated kinase
GTPɣ[^35^S], [^35^S]ɣ [guanosine-5′-O-(3-[^35^S]thio)triphosphate]; GSK1034702: 7-fluoro-5-methyl-3-[1-(oxan-4-yl)piperidin-4-yl]-1*H*-benzimidazol-2-one
IP: inositol phosphate
mAChR: muscarinic acetylcholine receptor
NMS: *N*-methylscopolamine
PAM: positive allosteric modulator
TBPB: 1-[1′-(2-methylbenzyl)-1,4′-bipiperidin-4-yl]-1,3-dihydro-2*H*-benzimidazol-2-one
